# Effects of alpha-cyclodextrin on water transport, cell hydration and longevity

**DOI:** 10.18632/aging.202533

**Published:** 2021-01-19

**Authors:** Lajos Szente, István Puskás, Tibor Vellai, Horst Lohmann

**Affiliations:** 1 CycloLab, Cyclodextrin R&D Laboratory, Ltd, Budapest H-1097, Hungary; 2 Department of Genetics, Eötvös Loránd University, Budapest H-1117, Hungary; 3 MTA-ELTE Genetics Research Group, Budapest H-1117, Hungary; 4 Ecocyte Bioscience, Dortmund D-44379, Germany

**Keywords:** cyclodextrin, cellular hydration, aquaporin, life span

## Abstract

Among parent cyclodextrins (CDs), alpha-CD (a-CD) has been utilized in a number of nutraceutical products, and approved as a dietary fiber to affect glycemic response and reduce dietary fat absorption. To extend our current knowledge on the biology of this natural carbohydrate, here we investigated its potential effects on cellular water uptake and aging. Two independent *in vivo* biological test systems were used, a single cell *Xenopus* oocyte with expressed human aquaporin for cell hydration studies and the nematode *Caenorhabditis elegans* for testing life span in the treated animals. a-CD was found to enhance water uptake through aquaporins of oocytes. Furthermore, the compound promoted longevity in *C. elegans*. Together, these results raise a rational for assaying a-CD as a potent drug candidate in treating various age-related diseases.

## INTRODUCTION

Cyclodextrins (CDs) have long been reputed as non-toxic multifunctional excipients, and utilized mainly as solubilizers and stabilizers for lipophilic compounds [1]. The unique and tunable solubility of CDs have been utilized in various applications including drug-delivery, food and beverage stabilizers and emulsifiers, and odor maskers. Additionally, CDs can act as kosmotropes to increase the structure or order of surrounding solvent environments, and kosmotropes are currently used to stabilize proteins, such as enzymes [2]. These features present an opportunity to evaluate the potential of using CD as a protective chaperone that can protect against dehydration and increase water permeability.

Chemically modified CDs can be hydrophilic or relatively lipophilic based on their substitution, and this impacts the way in which they are able to act as permeability enhancers [3]. Lipophilic CDs, such as the methylated derivatives, are thought to increase drug flux by reversibly altering the barrier properties of the membrane through selective component depletion or solubilization [4]. The hydrophilic CD family also modulates drug flux through membranes but via different mechanisms.

The solubility of CDs in water is unusual, and shows a rather unique, irregular trend. Beta-CD (b-CD), also called cyclomaltoheptaose, is about an order of magnitude less soluble in water than the cyclomaltohexaose (a-CD) and cyclomaltooctaose gamma-CD (g-CD). This intriguing behavior has been investigated, and some interesting explanations regarding the effect that CD can have on the lattice structure of the surrounding water have been proposed. Sabadini et al [5] compared the solubility of the three above natural CDs in H_2_O and D_2_O, revealing a much lower solubility of all the three CDs in heavy water. The authors propose that this is a result of the differences in hydrogen bonds that form between the different CDs and H_2_O and D_2_O. CDs can act as kosmotropes to change the local water structure, and due to the hydrophobic effect where D_2_O forms stronger hydrogen bonds as compared to H_2_O, the entropic–enthalpic balance involved in dissolution of the CDs is more unfavorable in D_2_O, resulting in the solubility of CDs is significantly higher in H_2_O, in comparison to their solubility in D_2_O [5].

CDs have been shown to exist as aggregates in water solutions, bound together by a network of hydrogen bonds [6]. Loftsson et al [7] demonstrated that dissolved parent CDs have a tendency to self-assemble and form different sized nano- and micro-aggregated particles in water, bound together by intermolecular H-bonds. Not only CDs, but also their non-covalent inclusion complexes show this phenomenon.

Some carbohydrates, such as saccharose and trehalose, have long been utilized to positively affect cellular water uptake and hydration processes in living organisms [8]. Trehalose has been thoroughly studied, and shown to cause structural changes in H-bonding and clustering of water molecules in liquid water [9]. This effect is often utilized to protect sensitive molecules from the physical shock during freezing, thawing and dehydration procedures, as well as in dry preservation of biologicals samples like cells, tissues and organs. Weng et al. [10] reported the effects of water on the structure and dynamics of trehalose glasses at low water contents and its relationship to preservation efficacy. Another study discussed the effect of trehalose and sucrose on the hydration and dipole potential of lipid bilayers mimicking cell membranes [11]. The water activity in dimyristoylphosphatidylcholine phospholipid bilayer (DMPC) has been shown to decrease by 60% when the lipid is dehydrated in the presence of trehalose at concentrations higher than 0.02 M [11]. In contrast, sucrose at 10 times higher concentrations produced only a 20% decrease in the water activity of the sample. Titrations of a DMPC solution in chloroform yielded 14 water molecules per lipid when pure water was added and seven water molecules per lipid when titrated with 0.025 M trehalose.

All living organisms must be able to regulate the flow of water into and out from cells, as the free movement of water across cell membranes is fundamental to life. The regulation of water transport across cell membranes is governed by special water channel proteins termed aquaporins. Aquaporins are found in many forms of life, including fungi, plants and animals [12, 13]. It is commonly accepted that most of the membrane-related transport processes are affected by both membrane physical properties, such as fluidity, lipid packing density, transepithelial electric resistance, etc., and chemical composition of the respective membrane domain. The mutual interactions between the membrane-integrated water channels and membrane components around these aquaporins were studied in detail by Martinez-Ballesta et al. [14]. This study concluded that the phospholipid membrane constituents comprised from saturated fatty acids are related to low membrane fluidity, high micro-viscosity, and low aquaporin functionality. On the other hand, the membrane domains-containing phospholipids with unsaturated fatty acids and sterols (such as cholesterol) enhance membrane fluidity and favor the open state and high functionality of aquaporins. It has been reported that a-CD preferentially interacts with these latter types of phospholipids, and therefore hypothesized that through this mechanism it could enhance the functionality of aquaporin [15].

The objective of this study was to assess the potential for a-CD to act analogously to trehalose as a chaperone to protect cells against desiccation, and to improve the hydrated status of cells. This was conducted by measuring water uptake in *Xenopus* oocyte cells with expressed human aquaporin. We also assayed its potential anti-aging effect in *C. elegans*, as trehalose promotes longevity in this organism [16]. Aging is driven by the lifelong, progressive accumulation of unrepaired cellular damage, mainly including unfolded, oxidized and aggregated proteins, which leads to disease and ultimately organismal death [17]. Because the rate at which cells age is largely determined by the activity of autophagy (cellular self-eating) [18–20], which functions as a major cell degradation process of eukaryotic cells to eliminate cellular damage, we also tested whether a-CD can modulate (enhance) the activity of autophagic degradation, and found that this compound exerts such positive effect.

## RESULTS AND DISCUSSION

### The physicochemical properties of a-CD-enabled water solutions

For technical and safety reasons, we investigated the effect of addition of low amounts of a-CD on the physical properties of bulk water. Previous studies indicated that a-CD aqueous solutions turned slightly hazy even at 2-5% concentrations, depending on the source and origin of a-CD (CycloLab, unpublished). Despite the good intrinsic solubility of a-CD in water the aqueous solutions remained unreliably clear transparent upon storage. This anomalous physical behaviour of a-CD solutions initiated studies on the diluted (between 0.05 %-2.0 % concentrations) a-CD solutions. We found that the presence of dissolved a-CD at their low concentration (0.05%) had a density-decreasing (kosmotropic) effect in purified water. At higher concentrations (0.5% and 1.0% solutions), however, this effect did not show up due to the higher solid content, which eventually caused an overall increase in the density of the bulk liquids. Clarity, pH, conductivity, density, viscosity, turbidity, surface tension and osmolality were determined in solutions prepared with purified water. The results are summarized in Table 1.

**Table 1:** Physico-chemical properties of test solutions containing a-CD vs. control purified water.

**Parameter**	**No additive**			**Alpha cyclodextrin**	
CD concentration	0.00%	0.05%	0.10%	1.00%	2.00%
pH	6.28	6.20	6.36	6.55	6.70
conductivity					
(μS·cm^-1^)	34.3	35.3	39.0	39.6	38.7
density (at 22 °C)					
(g·cm^-1^)	0.9980±0.0005	0.9900±0.0020	0.9890±0.0010	0.9930±0.0020	1.0480±0.0010
viscosity (25 C°)					
(cP)	0.91	0.91	0.92	0.97	1.01
Turbidity (Absorbance,					
λ= 410 nm)	(reference)	0.003	0.000	0.017	0.015
Visual inspection	clear	clear	clear	clear	clear
Surface tension					
(mN·m^-1^)	72	72	72	72	73
Osmolality					
(mOsm/kg)	0	0	2	8	18

Diffusion enhancing compounds that increase the specific volume of the aqueous system have been described in the literature [21], where an increase in water structure is achieved by affecting the extent and strength of hydrogen bonding among water molecules. Both the extent and strength of hydrogen bonding may be changed by a kosmotrope. We found that the presence of dissolved CDs at their low concentration (0.05%) has a density-decreasing (kosmotropic) effect in purified water. At higher concentrations (0.5% and 1.0% solutions), however, this effect did not show up due to the higher solid content, which eventually caused an overall increase in the density of the liquids.

### Biological testing of a-CD-containing aqueous solutions

This section deals with the observations collected by using two different biological model systems to assess how the presence of a-CD affect cellular hydration process. Two independent biological models were employed:

- water permeation test on the single-cell *Xenopus laevis* frog oocyte
- life span test on the multicellular nematode *Caenorhabditis elegans*

### Effect of CDs on cell hydration, using Xenopus frog oocytes

Rationale of Xenopus frog oocyte test

Water represents about 70-90% of the weight in a living cell. Water can cross lipid bilayers of biological membranes by diffusion. However, diffusion is a slow, non-regulated process which cannot account for the selective transmembrane water permeation that occurs in many fluid transporting tissues during normal physiological processes, suggesting the existence of some other pathways for the cellular water uptake. The discovery of water channels called aquaporins gave an explanation for the rapid and regulated transport of water across the lipid bilayers of cell membranes. In our studies we used the same biological system and tested the method that was introduced by Peter Agre [12], making his ground-breaking discovery and describing the function of aquaporins. Frog oocytes (eggs) are normally resistant to water permeation, as mother frogs lay their eggs in water. Peter Agre injected ribonucleic acid into oocytes, resulting in expression of membrane-anchored water channel proteins allowing oocytes to become permeable for water. This experimental set up enables the investigation of the extent of water uptake through aquaporins of oocyte cells under different experimental conditions.

**Figure 1:**
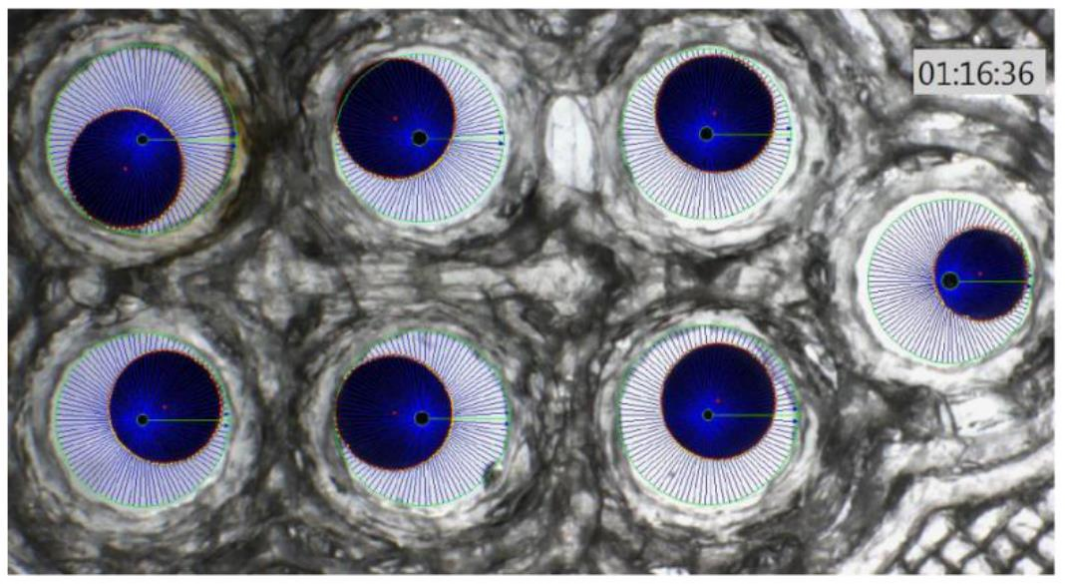
***Xenopus* oocyte chamber with 6 oocytes (blue spheres) with different swelling and a calibration oocyte on the right of the photo.**

**Figure 2:**
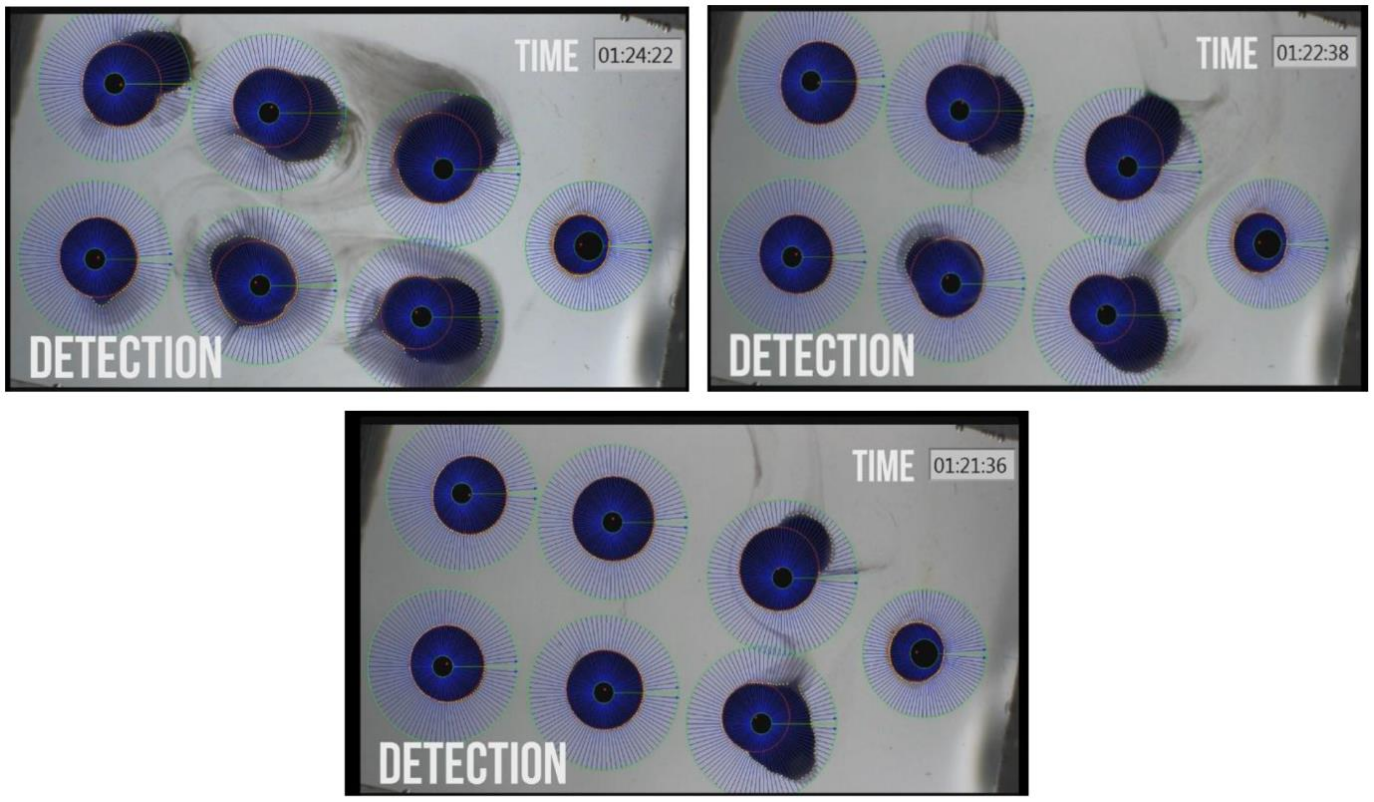
**Photographs of Xenopus oocyte chambers of 6 a-CD treated oocytes with different swelling due to water permeation into the cells and rupture of oocyte due to high volume expansion by water uptake.** Photos were taken at increasing time points of treatment. The control un-swollen small oocyte is on the right hand site of each photo.

In this experiment, *Xenopus* oocytes expressing human aquaporin 1 (AQP1) were subjected to different solutions of a-CD, and monitored for water uptake as compared to control, which was exposed to water only. To the best of our knowledge, this is the first time that an oocyte water channel testing method is used for the description of the effect of a CD on cellular water uptake through human AQP1. The results of oocyte osmotic water permeability over time are illustrated on Figures 1, 2 where each blue sphere represents an oocyte in a different aqueous solution, and a calibration oocyte on the far right.

**Figure 3:**
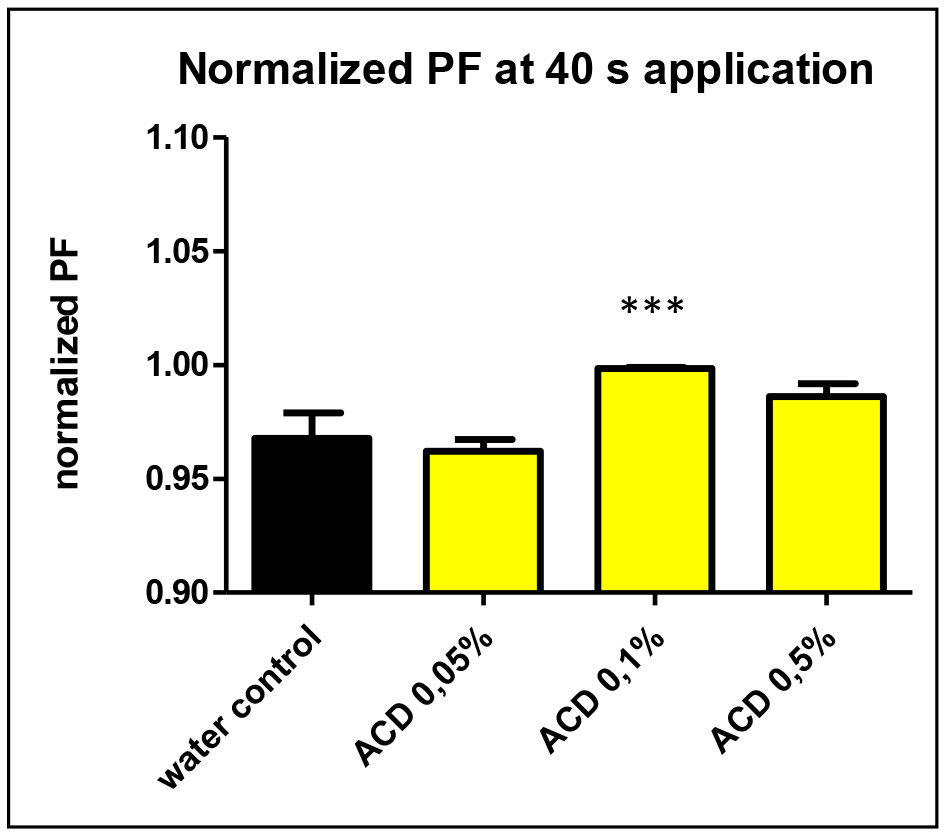
**The normalized osmotic water permeability (PF values) of aquaporin expressed oocytes at 40 sec.** **application of 0.05, 0.1 and 0.5 w/v% a-CD solutions vs. water control treatment**. T-Test results against water control: ACD 0.05% (P=0.11 n.s.), ACD 0.1% (P<0.0001 ***) ACD 0.5%: (P=0.17 n.s.).

Among the studied CD-enabled water samples, 0.1% a-CD solution was found to have significantly enhanced water uptake. This can be seen also on Figure 3. showing the normalized PF values at 40 seconds application.

Although the single cell oocyte studies were performed with a limited number of repetitions (N=6), the effect of 0.1 w/v% a-CD-containing water showed a significantly improved water uptake by cells. Further studies with greater number of repetitions on oocyte test are planned to support the first observations on CD-enhanced water.

### Effect of a-CD on C. elegans life span

*C. elegans*, of which around 50% of its genes possess significant human homologs, mainly exists as a self-fertilizing hermaphrodite that produces both oocytes and sperms. At 25° C, the worm’s reproduction cycle is relatively short (3 days only), and adult animals can live for approximately 2 weeks. Adult hermaphrodites produce eggs (embryos) for the first 5-6 days of adulthood, then become sterile and exist for an additional week. During this latter phase of adulthood, the animal progressively accumulates large vacuoles/lipid droplets in several tissues (mainly in intestinal cells), and become paralyzed. When animals die, they no longer respond to being touched by a platinum wire, whereas the living animal normally responds to a gentle touch stimulus to the nose by initiating a backward locomotion. Because of these tractable characteristics, *C. elegans* is a commonly applied multicellular genetic model system to study aging.

**Figure 4:**
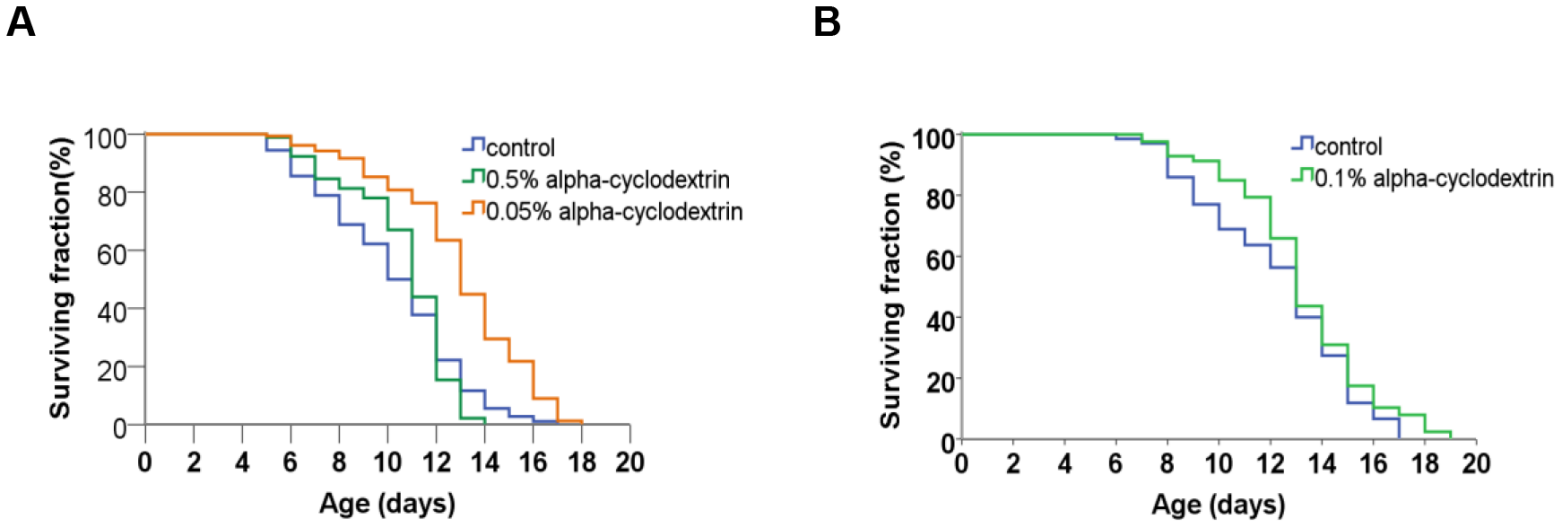
**a-CD treatment can extend life span in *C. elegans*.** (**A**) Kaplan-Meyer lifespan curve of control (untreated – blue curve) vs. a-CD-treated (0.05 w/v% - orange curve, 0.5 w/v% - green curve) animals. Animals treated with 0.05 w/v% a-CD live longer by 2 days on average than control. Both mean and maximum life spans become longer in response to treatment (see Supplementary Tables 1, 2). A higher concentration of a-CD (0.5 w/v%), however, does not lead to a longevity response. (**B**) a-CD at a working concentration of 0.1 w/v% slightly influences *C. elegans* life span. Kaplan-Meyer lifespan curves are shown. For statistics, see Supplementary Tables 1, 2.

Control, synchronised nematodes were maintained at 25° C on plastic plates (60-70 wild-type adult hermaphrodites/plate) containing standard nematode growth medium (NGM) prepared with tap water. Individuals were carefully transferred to new agar plates at each day until they stopped laying eggs. Experiments were performed in triplicates. The growth curve of these animals (see Figure 4, blue curves on panels A and B) was highly similar to those reported previously in the literature [22, 23]. a-CD-treated animals were cultured on NGM plates supplemented with different concentrations of a-CD. Animals that were treated with 0.05 w/v% solutions of a-CD exhibited a significantly extended lifespan as compared with control (untreated) ones (see Figure 4, orange curve on panel A). When applied at a higher concentration (0.5 w/v %), however, the lifespan-extending effect of a-CD was diminished (Figure 4, green curve on panel A). This may result from the fact that a relatively high concentration (0.5%) of a-CD hyperactivates a cellular pathway that normally contributes to longevity, and the exalted pathway may exert a pleiotropic effect (e.g., it slows down the rate at which cells age and, in parallel, causes a pathological condition). To test this possibility, a-CD was tested at 0.1% concentration, and treated animals indeed lived slightly longer than control (Figure 4 panel B). The data represented at Figure 4 panels A and B were recorded independently (i.e., not simultaneously), which gives an account to the control data variations. From these data we can conclude that a-CD significantly promotes longevity in this organism when used at a certain (optimal) concentration range.

**Figure 5:**
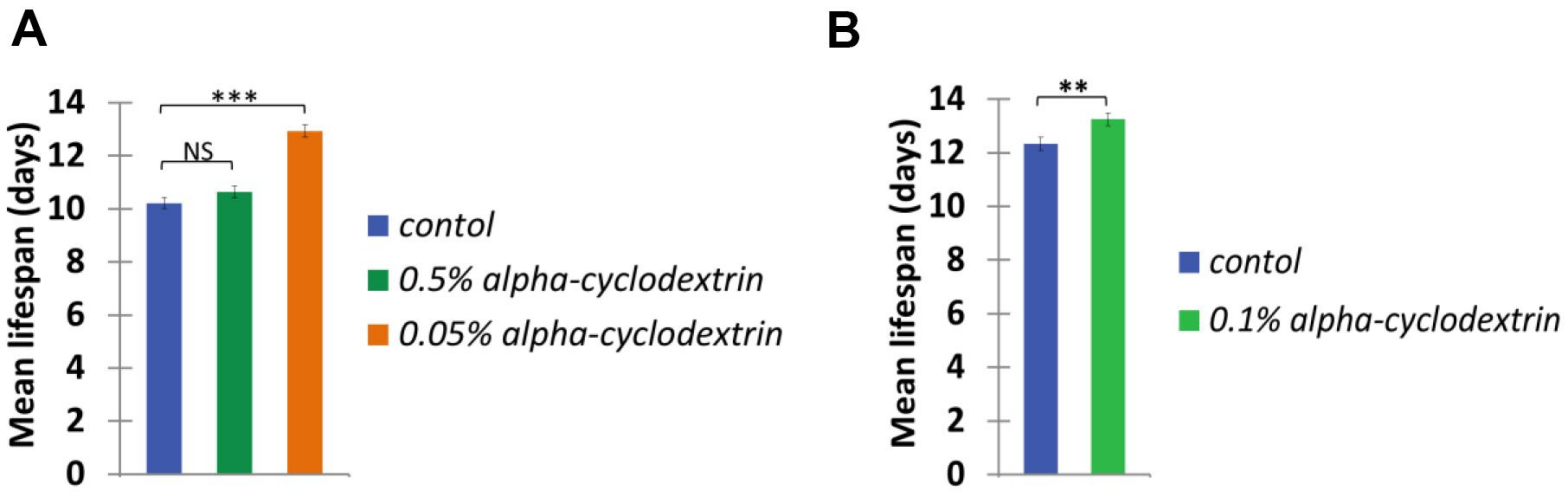
**a-CD extends *C. elegans* mean life span.** Mean life span of nematodes treated with 0.05, 0.50 w/v% (**A**) and 0.10 w/v% (**B**) a-CD-containing water samples.

It showed even more convincing longevity effects of the a-CD-enabled water, when the mean lifespan calculated from the Kaplan-Meyer lifespan curves data of the a-CD-treated worms is demonstrated (Figure 5). Again, the treatment with a relatively low concentration of a-CD (0.05 w/v%) provided the most significant longevity effect.

The data of statistical evaluation of the above C.*elegans* study are listed in Supplementary Tables 1, 2.

## CONCLUSIONS

a-CD-enabled water containing low concentrations (0.05-0.5 %) of a-CD was found to provide beneficial effects. Among many already published positive effects of a-CD consumption, we found that this carbohydrate also affects cellular water uptake and longevity, studied on two independent biological models. The single cell *Xenopus* oocyte with expressed human AQP1 was for the first time used to assess the a-CD facilitated water transport through water channels.

*C. elegans* was used for testing the anti-aging effect of a-CD, provided evidences on the lifespan-enhancing effect of a-CD applied at 0.05-0.5 w/v% concentrations. Additional work is planned to be performed to compare effects of different cyclodextrins to that of simple sugars.

In the past few decades, CDs have been successfully used for molecular encapsulation and as formulation enabling additives to improve the stability, aqueous solubility of lipophilic compounds. Around 60 pharmaceutical formulations are on the market containing CDs. During the last 15 years, however, some CDs have been found to act as pharmaceutical active agents, themselves opening a new era for CD applications. a-CD-enabled water containing low concentrations (0.05-0.5%) of a-CD was assessed for its ability to impact cell hydration and longevity using *Xenopus* oocyte and *C. elegans* assays, and was found to provide beneficial effects in both cases. *Xenopus* oocytes test is a useful model for studying water transport through aquaporin expression and *C. elegans* is widely used as a tractable ageing model, and results obtained from these models may be translated to human health and life span. Further studies are in progress to support these early observations, optimize the formulation and assess potential benefits of combining a-CD with other nutrients. The mechanism of action of these encouraging nutraceutical effects of a-CD treatments need to be explored with the aim of clarifying whether the demonstrated effects of a-CD is a direct or an indirect consequence of CD consumption.

Based on these results, we conclude that the CD formulation tested offers a significant improvement on a cell’s ability to hydrate via the aquaporin pathway and can increase the longevity of a multi-celled organism. It was also observed that the CD treated *C. elegans* showed a considerable increase in vitality during the mid-point of their lives. These results demonstrated that concentrations below 0.1% perform better compared to higher concentrations, and we believe that this is correlated with a decrease in aqueous density that was also observed. Results shown in the present study further support previous findings that some CDs, alone, possess beneficial pharmacological, nutraceutical properties, with their highly favorable safety profiles upon oral administration. These findings may initiate further investigations into the mechanism of action, how concentrations of a-CD affect aging and how a-CD enhances cellular hydration. The present results may also accelerate the study of the therapeutic utility of these unique carbohydrates in senescence and age-associated neurodegeneration, such as Alzheimer’s, Parkinsonism, type-2 diabetes, atherosclerosis, macular degeneration etc. [24]. Additional work to optimize the formulation to assess the potential benefits of combining other ingredients including those that may form complexing agents within the CD cavity should be performed, as well as evaluation in human subjects.

## MATERIALS AND METHODS

### Materials

a-CD (food grade, Cawamax W6) was the product of Wacker Chemie, Germany. As water sources, two types of water were used:

### Municipal tap water

Municipal tap water available at the site of study was used for *C. elegans* lifespan studies. The tap water had the characteristics listed in Table 2.

**Table 2:** Characteristics of municipal tap water used for *C. elegans* lifespan studies.

**Parameter**	**Value**
Free active chlorine	0.18 mg/l
Chloride	24 mg/l
Iron	6 μg/l
Manganese	2 μg/l
Nitrate	9 mg/l
Nitrite	<0.03 mg/l
Ammonium	<0.04 mg/l
Hardness of water	122 mg/l CaO
Osmolality	9 mOsm/kg
Conductivity	442 μS/cm
pH	8.0

### Purified water

Purified water was used for physico-chemical experiments. This solvent was produced by removal of dissolved ions from the municipal tap water available at the site of the studies by Merck/Millipore Synergy^®^ Water Purification System. The instrument produces Type 1 water (18.2 MΩ•cm at 25° C ultrapure water) on demand.

### Methods

### Physico-chemical characterization methods

Turbidity measurements (solution clarity test)

An UV/VIS photometer Agilent 8453 was used applying a 1 cm quartz cuvette. Absorbance at 410 nm wavelength was recorded.

pH measurement

Potentiometric pH measurement was applied using a combined glass electrode (Mettler Toledo InLab micro) and a Metler Toledo SevenCompact benchtop pH meter.

Conductivity

Conductivity was determined by Mettler-Toledo SG7 Conductivity meter equipped with Mettler-Toledo InLab 741 electrode at 25° C.

Viscosity measurements

Viscosity of test solutions was determined by Brookfield DV2T Viscometer at 25° C. Sample volume of 0.5 mL was used and spindle type CPA40Z was applied. Spindle speed was selected to result optimal torque of 25-75% built-in value depending on the actual viscosity.

Density measurements

Density of solutions was determined at 22° C in a 10 ml volumetric flask of A grade precision by measuring the weight of the solutions on an analytical balance of 1 mg precision.

Surface tension measurements

Surface tension was measured by stalagmometer (stalagmometric method) at 22° C. The surface tension of the test solution (γ_test_) can be calculated by counting the number of droplets formed at the bottom of the modified burette relative to distilled water of known surface tension (72 mN·m^−1^). The number of droplets of water (*n_water_*) and that of test solution (*n_test_*) are correlated with γ_test_ according to the following formula:

$$\gamma_{test}=72\;mN\cdot m^{-1}\frac{n_{water}}{n_{test}}$$

### Biological test systems

### Xenopus laevis oocyte test

Oocyte harvesting

To harvest oocytes, adult *Xenopus laevis* from Ecocyte Bioscience's own colony were anaesthetized with 0.15% MS-222 in water for 15 min. Then, animals were kept on ice for another 15 min before ovarectomy was performed. Ovaries were incubated in collagenase (Worthington Type II, 10 mg/ml) in calcium free Barth's solution (CFBS, 88 mM NaCl, 1 mM KCl, 0.8 mM MgSO_4_, 5 mM TRIS-HCl, 2.4 mM NaHCO_3_). Following defolliculation, oocytes were rinsed in normal Barth's solution (MBS, NaCl 88 mM, KCl 1 mM, CaCl_2_ 0.4 mM, Ca(NO_3_)_2_ 0.33 mM, MgSO_4_ 0.8 mM, TRIS-HCl 5 mM, NaHCO_3_ 2.4 mM) before they were transferred to 96-well plates. For nuclear injection of the different DNA's into the oocytes, the Roboocyte automated injection and recording system was used.

For cytoplasmic injection of the mRNA encoding human Aquaporin-1 channels into the oocytes, the Roboocyte automated injection and recording system was used.

Aquaporine 1 transcription

Human Aquaporine 1 cDNA cloned in expressing vector pGEM-T were purchased from Sino Biological Inc.. Transcription to AQP1 mRNA was performed by an Ecocyte cooperation partner lab (Inst. für Pharmaceut and Medical Chemistry, Univ. Muenster).

mRNA Injection

For injection of mRNA coding for the human AQP1 channel the Roboocyte automated injection and recording system was used. Injection volume was in the range 20-50 nl at a mRNA concentration of 100 ng/μl. After two to three days of incubation in Barth's solution supplemented with Gentamycin, water uptake of the *Xenopus* Oocytes through AQP1 channels was tested in a swelling assay using video microscopy.

Compound preparation

All test compound aqueous a-CD solutions with 0.05%, 0.1%, 0.5% concentrations were prepared in purified water. Normal frog ringer (NFR, NaCl 90 mM, KCl 2 mM, CaCl_2_ 2 mM, MgCl_2_ 1 mM, HEPES 5 mM, osmolarity 200 mOsm/l) was used as control solution and was prepared freshly on the day of the experiments. All solutions were handled in double blind experiments.

Swelling Assay/Video microscopy

For video microscopy after 2 days of expression time, *Xenopus* oocytes expressing the AQP1 channel were transferred into a specific Oocyte chamber fitting 6 *Xenopus* oocytes at a time filled with NFR at room temperature for 15 min. NFR was then exchanged to one of the unknown solutions C1-C10 for further 45 min. To control the water uptake, the size of oocytes was controlled with a video camera equipped with a microscopic objective during the complete experiment of 60 min, and saved on hard disc for a later offline analysis. From the 60 min video recording, serial images were taken at an interval of 2 s during the 15 control period (superfusion with NFR) and an interval of 2 s during the 45 min of compound superfusion.

Data analysis

The size of *Xenopus* oocytes was measured by use of an image processing software (Ecocyte Bioscience) from the serial images. The software measures the diameters of oocytes in 2 main axis and calculates the surface area (S) and the volume (V) out of these measures. The osmotic water permeability (Pf) was then calculated according the following formula:

P_f_ = (V_0_ x (d(V/V_0_)/dt))/(S x V_w_ x (Osm_in_-Osm_out_))

with V_w_ being the molar volume of water (V_w_=18 cm^3^/mol).

V_0_ = the volume of the Oocytes directly before switching the solution from control NFR to the test solution (C1-C10)

d(V/V_0_)/dt = the increase of the oocyte volume over time

S = surface area of oocytes

Osm_in_-Osm_out_ = difference in the osmolarity of the control solution to the test solution; = 192 x 10^-9^ mol/mm^3^

From these values the time courses of the osmotic water permeability P_f_ were calculated.

#### Lifespan assays

Bristol (N2) was used as wild-type *C. elegans* strain in this study. Nematodes were maintained and propagated on Nematode Growth Medium- (NGM) containing plates, and fed with *Escherichia coli OP50* bacteria. The preparation of NGM agar plates is detailed elsewhere (http://www.wormbook.org/chapters/www_strainmaintain/strainmaintain.html#d0e214.)

Lifespan assays were carried out at 25° C. For synchronization, 20-30 gravid, well-fed adult hermaphrodites were transferred to a new NGM agar plate to lay embryos for 4-5 h, then removed. Approximately 60-70 F1 young (non-gravid) adults were transferred individually to new NGM plates supplemented with different substances examined (t=0). F1 adults were then assayed for survival. Animals were individually transferred to new fresh plates by a platinum wire at each day until they stop to lay eggs (to avoid mixing the subsequent generations). Animals that climbed up the wall of plastic dishes or exhibited a protruded vulva phenotype were excluded from the analysis. Animals were considered dead when they stopped pharyngeal pumping and responding to touching. SPSS 17 software was used to calculate mean lifespan and perform statistical analysis. P values for comparing Kaplan-Meyer survival curves between two groups were determined using log-rank (Mantel-Cox) tests, and P values for comparing mean lifespans were determined using Independent samples *t*-tests with Bonferroni correction.

## Supplementary Material

Supplementary Tables
