# Supplementary material for: Effects of alpha-cyclodextrin on water transport, cell hydration and longevity
Source: Aging (Albany NY). 2021 Jan 19;13(2):1718–28. doi: 10.18632/aging.202533 (PMC7880385; doi:10.18632/aging.202533)
Supplement: Supplementary Tables [file aging-13-202533-s001.pdf]

SUPPLEMENTARY TABLES

Supplementary Table 1. Statistics for lifespan data seen in Figure 4A wild-type hermaphrodite animals were tested.

	Number of worms	Mean lifespan (days)	SD (days)	SEM (days)	Log rank p value (with bonferroni correction)	Kruskal-wallis H test with Dunn's Post hoc test P value
control	180	10.21	2.90	0.216		
0.5 w/v% alpha-cyclodextrin	91	10.64	2.18	0.228	vs control p=2.616	vs control p=1.000
0.05 w/v% alpha-cyclodextrin	156	12.94	2.88	0.230	vs control p<0.000	vs control p<0.000

Supplementary Table 2. Statistics for lifespan data seen in Figure 4B wild-type hermaphrodite animals were tested.

	Number of worms	Mean lifespan (days)	SD (days)	SEM (days)	Log rank p value	Independent T-test p value
control	135	12.33	2.90	0.249		
0.1 w/v% alpha-cyclodextrin	126	13.25	2.68	0.238	p=0.025	p=0.009
